# Nonsteroidal Antiinflammatory Drugs and Group A Streptococcal Infection

**DOI:** 10.3201/eid1208.051067

**Published:** 2006-08

**Authors:** David M. Aronoff, Zuber D. Mulla

**Affiliations:** * University of Michigan Health System, Ann Arbor, Michigan, USA;; † University of Texas School of Public Health at Houston, El Paso, Texas, USA

**Keywords:** group A streptococcus, nonsteroidal antiinflammatory drugs, protopathic bias, letter

To the Editor: Factor et al. recently reported the results of a population-based, case-control study regarding risk factors for pediatric invasive group A streptococcal (GAS) infection (*1*), noting that the "new" use of nonsteroidal antiinflammatory drugs (NSAIDs), defined as NSAID use <2 weeks before diagnosis, was associated with invasive GAS infection, whereas self-defined "regular" NSAID use was not. The control population consisted of nonhospitalized, age-matched children contacted by telephone (*1*). Although we endorse the authors' conclusion that, "…the measurements of new use and regular use [of NSAIDs] are too crude to clearly identify their role as a risk factor," a more detailed discussion of their findings and conclusions is warranted.

Because of their antiinflammatory effects, NSAIDs have been suspected of suppressing host immunity during infection, particularly GAS infection (*2*). However, determining a causal association between NSAID use and infectious diseases has been problematic, especially when using retrospective studies (*3*). The results of such observational studies often suffer from protopathic bias, in which drugs are applied to treat symptoms that are actually early manifestations of the outcome of interest (*4*). Consequently, rather than being a direct determinant (i.e., causative risk factor) for invasive GAS infection, NSAID use could mark the onset of disease symptoms (fever, localized pain, and inflammation). Therefore, because of protopathic bias, the study by Factor et al. had a substantial chance of identifying an association between NSAID use and invasive GAS infection a priori.

Neither the fact that patients in the study by Factor et al. received NSAIDs any time during the 2 weeks before the diagnosis of invasive GAS infection nor the finding that nonhospitalized children (controls) were unlikely to have received NSAIDs in the 2 weeks before their interview should be surprising. A more informative case-control study would have matched case-patients with similar-aged children who had febrile infections not caused by GAS infection; both groups of children would have been equally likely to have received analgesic and antipyretic medications. Furthermore, population-based data suggest that most patients with invasive GAS infection are hospitalized (*5*), so hospital-based controls, rather than population controls, might have provided a more appropriate comparison group.

Prospective studies have failed to define a causal link between NSAIDs and invasive GAS infections (*3*), though such studies were not specifically designed to investigate this relationship. To best test the hypothesis that NSAIDs increase the risk for invasive GAS infection, a randomized, prospective trial should be done. Such a trial is unlikely to take place, however, because of questionable ethics and because the sample necessary to detect a significant difference would be prohibitively large.

Although NSAIDs may neither alter the risk of developing an invasive GAS infection nor accelerate an established infection, these drugs can mollify the signs and symptoms of streptococcal infection, possibly delaying appropriate management and treatment (*3*). However, the potential adverse consequences of suppressing clinical indicators of disease severity (e.g., fever, pain, and inflammation) with NSAIDs apply to myriad infectious and inflammatory conditions, not just invasive streptococcal disease.
